# Crystal structure of the pyridine–diiodine (1/1) adduct

**DOI:** 10.1107/S2056989015010518

**Published:** 2015-06-13

**Authors:** Matti Tuikka, Matti Haukka

**Affiliations:** a University of Jyvaskyla, Department of Chemistry, P.O. Box 35, FI-40014 University of Jyvaskyla, Finland

**Keywords:** pyridine, diiodine, halogen bonding, crystal structure

## Abstract

In the title adduct, C_5_H_5_N·I_2_, the N—I distance [2.424 (8) Å] is remarkably shorter than the sum of the van der Waals radii. The line through the I atoms forms an angle of 78.39 (16)° with the normal to the pyridine ring.

## Related literature   

For the structure of the pyridine–I_2_ 1:2 adduct, see: Hassel & Hope (1961 ▸). For the crystal structures of pyridine with ICl and IBr, see: Rømming (1972 ▸); Dahl *et al.* (1967 ▸). For van der Walls radii, see: Bondi (1964 ▸). For the I—I distance of iodine, see: Buontempo *et al.* (1997 ▸). For I—I^⋯^N angles in halogen bonding, see: Desiraju *et al.* (2013 ▸).
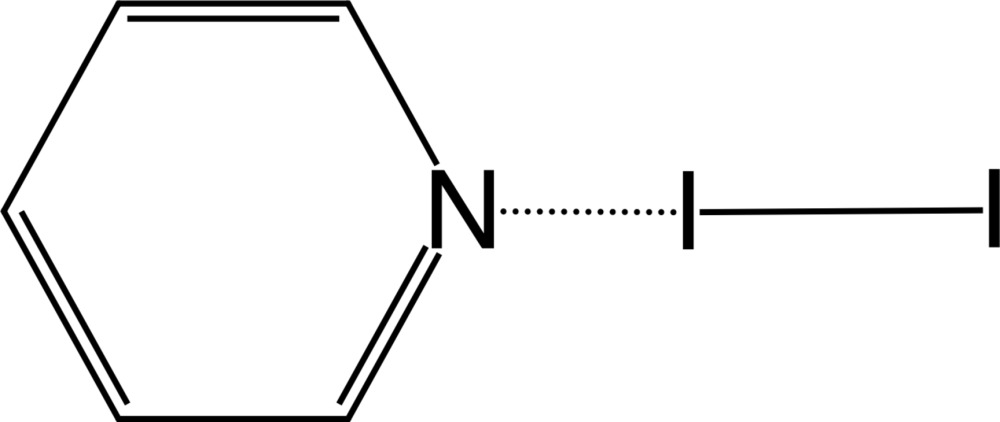


## Experimental   

### Crystal data  

C_5_H_5_N·I_2_
*M* *_r_* = 332.90
Monoclinic, 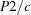

*a* = 9.2432 (6) Å
*b* = 4.3392 (2) Å
*c* = 20.1953 (13) Å
β = 98.468 (3)°
*V* = 801.16 (8) Å^3^
*Z* = 4
Mo *K*α radiation
μ = 7.76 mm^−1^
*T* = 120 K
0.09 × 0.07 × 0.02 mm

### Data collection  

Bruker KAPPA APEX II CCD diffractometer
Absorption correction: numerical (*SADABS*; Bruker,2012 ▸) *T*_min_ = 0.574, *T*_max_ = 0.902
6585 measured reflections
1853 independent reflections
1437 reflections with *I* > 2σ(*I*)
*R*_int_ = 0.062

### Refinement  

*R*[*F* ^2^ > 2σ(*F* ^2^)] = 0.049
*wR*(*F* ^2^) = 0.091
*S* = 1.07
1853 reflections
73 parameters
H-atom parameters constrained
Δρ_max_ = 1.11 e Å^−3^
Δρ_min_ = −1.26 e Å^−3^

### 

Data collection: Collect (Nonius, 2000 ▸); cell refinement: *DENZO*/*SCALEPACK* (Otwinowski & Minor, 1997 ▸); data reduction: *DENZO*/*SCALEPACK*; program(s) used to solve structure: *SUPERFLIP* (Palatinus & Chapuis, 2007 ▸; Palatinus & van der Lee, 2008 ▸; Palatinus *et al.*, 2012 ▸); program(s) used to refine structure: *SHELXL97* (Sheldrick, 2008 ▸); molecular graphics: *OLEX2* (Dolomanov *et al.*, 2009 ▸); software used to prepare material for publication: *OLEX2*.

## Supplementary Material

Crystal structure: contains datablock(s) I. DOI: 10.1107/S2056989015010518/rz5157sup1.cif

Structure factors: contains datablock(s) I. DOI: 10.1107/S2056989015010518/rz5157Isup2.hkl

Supporting information file. DOI: 10.1107/S2056989015010518/rz5157Isup3.cml

**Fig. 1.**
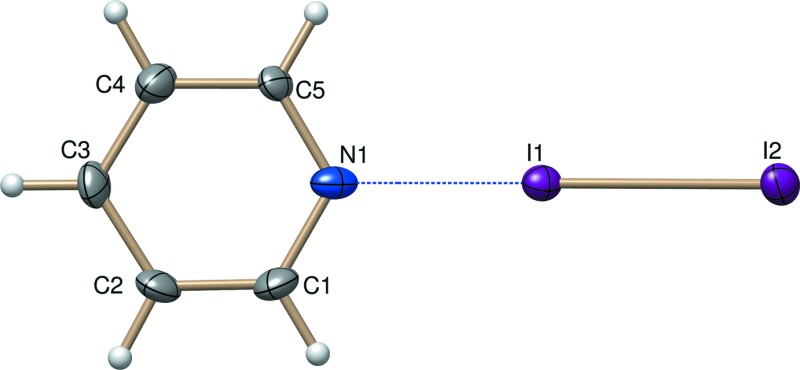
The molecular structure of the title compound, with 50% probability displacement ellipsoids for non-H atoms.

. DOI: 10.1107/S2056989015010518/rz5157fig1.tif
The mol­ecular structure of the title compound, with 50% probability displacement ellipsoids for non-H atoms.

CCDC reference: 1404151

Additional supporting information:  crystallographic information; 3D view; checkCIF report

## supplementary crystallographic information

### S1. Comment

Diiodine is capable to act as halogen bond donor and form stable halogen bonds with Lewis bases, such as pyridine, due to the strong charge transfer. In the case of the pyridine-I_2_ 1:2 adduct (Hassel & Hope, 1961), the interaction eventually results in the heterolytic cleavage of I_2_ and formaton of [py_2_I]^+^ I_3_^-^ ion pairs. Although the crystal structures involving pyridine and interhalogens ICl and IBr are known (Rømming, 1972; Dahl *et al.*, 1967), the title pyI_2_ 1:1 adduct has not been reported earlier. The N1—I1 distance in pyI_2_ (2.425 (8) Å) is remarkably shorter than the sum of the van der Walls radii of iodine and nitrogen (3.53 Å; Bondi, 1964). The I—I distance (2.8043 (9) Å) is significantly longer than that observed in free diiodine in solid state (2.715 Å; Buontempo *et al.*, 1997). The I—I···N angle is approximately linear (176.44 (18)°) as expected in halogen bonds (Desiraju *et al.*, 2013).

### S2. Experimental

The title compound was synthesized by dissolving iodine (200 mg) in ethanol (5 ml) and adding pyridine (1 ml) into this solution. The solution was left to evaporate unde ambient conditions and after a couple of days light yellow crystals were formed.

### S3. Refinement

All H atoms were positioned geometrically and refined using a riding model with C—H = 0.95 Å and with *U*_iso_(H) = 1.2 *U*_eq_(C).

### Crystal data

**Table:**

C_5_H_5_N·I_2_	*F*(000) = 592
*M**_r_* = 332.90	*D*_x_ = 2.760 Mg m^−^^3^
Monoclinic, *P*2/*c*	Mo *K*α radiation, λ = 0.71073 Å
*a* = 9.2432 (6) Å	Cell parameters from 1865 reflections
*b* = 4.3392 (2) Å	θ = 1.0–27.5°
*c* = 20.1953 (13) Å	µ = 7.76 mm^−^^1^
β = 98.468 (3)°	*T* = 120 K
*V* = 801.16 (8) Å^3^	Plate, clear light yellow
*Z* = 4	0.09 × 0.07 × 0.02 mm

### Data collection

**Table:**

Bruker KAPPA APEX II CCD diffractometer	1853 independent reflections
Radiation source: fine-focus sealed tube	1437 reflections with *I* > 2σ(*I*)
Curved graphite crystal monochromator	*R*_int_ = 0.062
Detector resolution: 16 pixels mm^-1^	θ_max_ = 27.6°, θ_min_ = 2.2°
φ scans and ω scans with κ offset	*h* = −11→11
Absorption correction: numerical (*SADABS*; Bruker,2012)	*k* = −5→5
*T*_min_ = 0.574, *T*_max_ = 0.902	*l* = −26→25
6585 measured reflections	

### Refinement

**Table:**

Refinement on *F*^2^	Primary atom site location: iterative
Least-squares matrix: full	Hydrogen site location: inferred from neighbouring sites
*R*[*F*^2^ > 2σ(*F*^2^)] = 0.049	H-atom parameters constrained
*wR*(*F*^2^) = 0.091	*w* = 1/[σ^2^(*F*_o_^2^) + (0.0153*P*)^2^ + 9.3396*P*] where *P* = (*F*_o_^2^ + 2*F*_c_^2^)/3
*S* = 1.07	(Δ/σ)_max_ = 0.001
1853 reflections	Δρ_max_ = 1.11 e Å^−^^3^
73 parameters	Δρ_min_ = −1.26 e Å^−^^3^
0 restraints	

### Special details

**Table:**

Geometry. All e.s.d.'s (except the e.s.d. in the dihedral angle between two l.s. planes) are estimated using the full covariance matrix. The cell e.s.d.'s are taken into account individually in the estimation of e.s.d.'s in distances, angles and torsion angles; correlations between e.s.d.'s in cell parameters are only used when they are defined by crystal symmetry. An approximate (isotropic) treatment of cell e.s.d.'s is used for estimating e.s.d.'s involving l.s. planes.

### Fractional atomic coordinates and isotropic or equivalent isotropic displacement parameters (Å^2^)

**Table:**

	*x*	*y*	*z*	*U*_iso_*/*U*_eq_	
I1	0.27234 (6)	0.59684 (13)	0.54587 (3)	0.02083 (16)	
I2	0.32645 (6)	0.35101 (14)	0.67558 (3)	0.02480 (18)	
N1	0.2243 (7)	0.8407 (18)	0.4368 (4)	0.0243 (18)	
C5	0.3349 (9)	0.933 (2)	0.4053 (4)	0.0207 (19)	
H5	0.4323	0.8743	0.4223	0.025*	
C3	0.1668 (10)	1.198 (2)	0.3214 (5)	0.028 (2)	
H3	0.1471	1.3178	0.2818	0.033*	
C1	0.0849 (10)	0.921 (2)	0.4121 (5)	0.030 (2)	
H1	0.0069	0.8529	0.4342	0.035*	
C2	0.0549 (10)	1.098 (2)	0.3558 (5)	0.030 (2)	
H2	−0.0434	1.1533	0.3399	0.036*	
C4	0.3076 (10)	1.112 (2)	0.3480 (5)	0.030 (2)	
H4	0.3872	1.1783	0.3266	0.036*	

### Atomic displacement parameters (Å^2^)

**Table:**

	*U*^11^	*U*^22^	*U*^33^	*U*^12^	*U*^13^	*U*^23^
I1	0.0189 (3)	0.0224 (3)	0.0212 (3)	−0.0010 (2)	0.0027 (2)	−0.0016 (3)
I2	0.0253 (3)	0.0267 (3)	0.0222 (4)	0.0029 (2)	0.0031 (3)	0.0011 (3)
N1	0.016 (4)	0.032 (4)	0.024 (4)	−0.003 (3)	0.004 (3)	−0.007 (4)
C5	0.017 (4)	0.031 (5)	0.013 (5)	−0.001 (4)	−0.001 (3)	0.000 (4)
C3	0.031 (5)	0.035 (6)	0.014 (5)	−0.002 (4)	−0.003 (4)	0.004 (4)
C1	0.019 (5)	0.039 (6)	0.031 (6)	−0.009 (4)	0.007 (4)	0.005 (5)
C2	0.014 (4)	0.049 (7)	0.026 (6)	−0.002 (4)	−0.003 (4)	0.002 (5)
C4	0.025 (5)	0.042 (6)	0.022 (6)	−0.006 (4)	0.005 (4)	0.003 (5)

### Geometric parameters (Å, º)

**Table:**

I1—I2	2.8043 (9)	C5—C4	1.388 (13)
I1—N1	2.425 (8)	C3—C2	1.397 (12)
N1—C5	1.342 (10)	C3—C4	1.383 (13)
N1—C1	1.357 (12)	C1—C2	1.364 (14)
			
N1—I1—I2	176.44 (18)	C4—C3—C2	116.6 (9)
C5—N1—I1	120.7 (6)	N1—C1—C2	121.1 (8)
C5—N1—C1	119.8 (8)	C1—C2—C3	120.9 (9)
C1—N1—I1	118.9 (6)	C3—C4—C5	121.2 (8)
N1—C5—C4	120.3 (8)		
			
I1—N1—C5—C4	−170.3 (7)	C5—N1—C1—C2	−0.8 (15)
I1—N1—C1—C2	170.4 (8)	C1—N1—C5—C4	0.8 (14)
N1—C5—C4—C3	−0.9 (15)	C2—C3—C4—C5	1.0 (15)
N1—C1—C2—C3	1.0 (16)	C4—C3—C2—C1	−1.0 (15)
