# Supplementary figures and images for: Crystal structure of the pyridine–diiodine (1/1) adduct
Source: Acta Crystallogr E Crystallogr Commun. 2015 Jun 13;71(Pt 7):o463. doi: 10.1107/S2056989015010518 (PMC4518975; doi:10.1107/S2056989015010518)

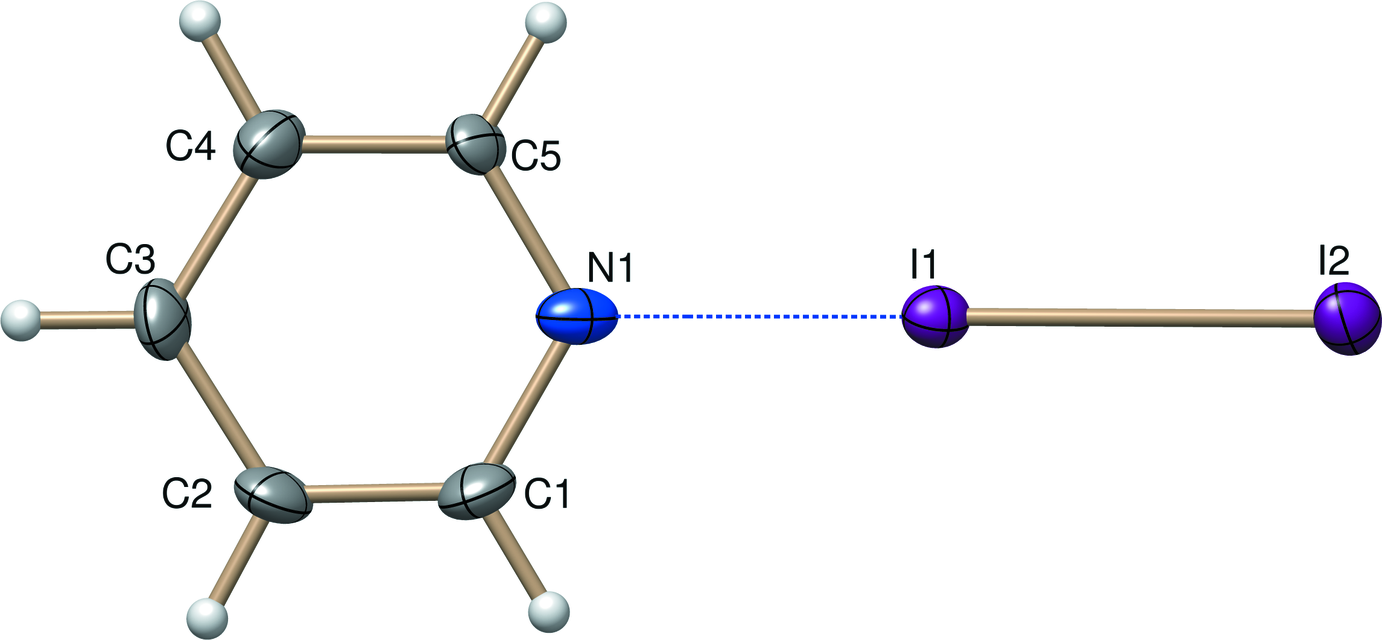

Supplement: Supplementary file 4 [file e-71-0o463-fig1.tif]
